# Effect of Bioactive Filler Addition on the Mechanical and Biological Properties of Resin-Modified Glass Ionomer

**DOI:** 10.3390/ma16051765

**Published:** 2023-02-21

**Authors:** Ana Carolina Diniz, José Bauer, Suzanni do Amaral Rodrigues Veloso, César Augusto Abreu-Pereira, Ceci Nunes Carvalho, Tarcísio Jorge Leitão, Leily Macedo Firoozmand, Etevaldo Matos Maia-Filho

**Affiliations:** 1 Dentistry Biomaterials Laboratory (Biomma), School of Dentistry, Federal University of Maranhão (UFMA), São Luis 65080-805, MA, Brazil; 2 Department of Postgraduate Program in Dentistry, CEUMA University, São Luis 65075-120, MA, Brazil

**Keywords:** tooth demineralization, glass ionomer cements, dentin

## Abstract

The maintenance of affected dentin can promote the greater conservation of tooth structure. The development of materials that have properties capable of reducing the demineralizing potential and/or even helping in dental remineralization is important for conservative dentistry. This study aimed to evaluate, in vitro, the alkalizing potential, fluoride as well as calcium ion release ability, antimicrobial activity, and dentin remineralization properties of resin-modified glass ionomer cement (RMGIC) incorporated with a bioactive filler (niobium phosphate (NbG) and bioglass (45S5)). The study samples were grouped into RMGIC, NbG, and 45S5. The materials’ alkalizing potential, ability to release calcium as well as fluoride ions, and antimicrobial properties concerning *Streptococcus mutans* UA159 biofilms were analyzed. The remineralization potential was evaluated using the Knoop microhardness test, which was performed at different depths. The alkalizing and fluoride release potential was higher for the 45S5 group (*p* < 0.001) over time. An increase in the microhardness of demineralized dentin was observed in the 45S5 and NbG groups (*p* < 0.001). No differences in biofilm formation were observed between the bioactive materials, although 45S5 exhibited lower biofilm acidogenicity at different time points (*p* < 0.001) and greater calcium ion release in the microbial environment. A resin-modified glass ionomer cement enriched with bioactive glasses, particularly 45S5, is a promising alternative for the treatment of demineralized dentin.

## 1. Introduction

Strategies aimed at the preservation of dental structures have been continuously studied, with the remineralization of partially demineralized dentin being verified [1], which can occur in the presence of a healthy pulp. There is also considerable evidence in support of the removal of infected dentin and the maintenance of affected dentin for the preservation of dental integrity [2]; additionally, the role of materials that protect the pulp–dentin complex and stimulate dentin remineralization as part of the repair process has been explored [3].

Conventional glass ionomer cement (GIC) is known as acid–base cement, based on the product of the reaction of weak polymeric acids with powered glasses [4,5]. The polymer (acid) attacks the glass (base) at its surface; in this way, the eruption of Ca^2+^ is followed by Al^3+^, among other reactive changes to the dynamics and material structure (Song et al. [5]). GIC is the material of choice for atraumatic restorative treatments and the modification of the oral environment through the release of fluorides for the prevention of carious lesion development [6]. Resin components, such as 2-hydroxyethyl methacrylate (HEMA) and the initiator (camphorquinone), have been incorporated into the same essential components as conventional glass ionomers (basic glass powder, water, and polyacid) [4], improving the mechanical properties of the resin-modified glass ionomer (RMGIC) [7]. RMGIC restorations are the result of the twin processes of neutralization (acid–base reaction) and addition polymerization. These two network-forming reactions may jeopardize the reliability of the set material [4,5]. The fluoride ions released by resin-modified GIC (RMGIC) can undergo a significant reduction 48 h after insertion, although the evidence on this is controversial [8]. The use of restorative materials with alkalizing potential and the ability to release fluoride [9] as well as induce remineralization [10] can contribute to the conservation of affected dentin, and ongoing research is largely focused on the identification of such materials to promote greater tissue remineralization.

In this context, bioactive materials have been studied extensively [11,12] due to their ability to penetrate the dentin structure, stimulate hydroxyapatite formation [12,13], and increase the pH of the medium [14]. Bioglass (45S5) and niobium phosphate (NbG) have been shown to interact with demineralized dentin [15], with the former exhibiting the ability to release large volumes of calcium phosphate ions that can transform into hydroxyapatite precursors [12,13]. NbG is an experimental bioactive glass that presents Nb–O bonds that are less permeable due to stronger chemical bonds, giving more stability to the material when compared to the bonds formed by silanol (Si–OH) groups. The high chemical stability and biocompatibility contribute to the slow deposition of ions [16] and increase microhardness [17] as well as antimicrobial action [16] without impairing mechanical properties [18]. These properties can contribute to limiting the progression of recurrent carious lesions in demineralized dentin [19], suggesting that the incorporation of bioactive materials in RMGIC can potentially improve its properties further.

The available literature shows that the incorporation of bioactive glasses (45S5 and NbG) into RMGIC can contribute to the alkalization of an acid medium (pH of 4.0) and promote greater fluoride as well as calcium ion release while preserving the mechanical properties (e.g., microhardness) of the material [18]; however, few studies have evaluated their interaction with and therapeutic effects on demineralized dentin when using treatment methods such as indirect pulp capping.

Thus, the objective of this in vitro study was to evaluate the effects of the incorporation of 10% 45S5 and NbG into RMGIC on its alkalizing potential and ability to release fluoride as well as calcium ions, remineralize dentin, and exert antimicrobial effects in a biofilm formed of *Streptococcus mutans* at different time points. The null hypotheses being tested were that no variations would be observed in (1) the alkalizing potential of the material and its ability to release fluoride ions at different time points; (2) the Knoop microhardness value of treated dentin at different depths (10, 50, 100, and 200 µm) and time points (24 h as well as 7, 30, and 60 days); (3) the antimicrobial activity of the material; and (4) the ability to release calcium ions in culture media.

## 2. Materials and Methods

### 2.1. Preparation of Bioactive Glasses

Niobiophosphate bioactive glass (NbG) (41.8% Nb_2_O_5_; 32.5% P_2_O_5_; 18.8% CaO; 2.7% Al_2_O_3_; 1.2% Na_2_O; and 0.04% SrO) was prepared using procedures reported previously [18,19], while 45S5 glass (45% SiO_2;_ 24.5% Na_2_O; 24.5% CaO; and 6% P_2_O_5_) (Sylc, Osspray Ltd., London, UK) was purchased commercially. The bioactive glasses were taken to a planetary mill (Pulveriette 5, Fritsch GmbH, Idar-Oberstein, Germany) for 30 min [11].

An analysis of the particle size (Mastersizer 2000, Malvern Instruments, Malvern, UK) of the material was performed, and representative images of the bioactive glass were created using a scanning electron microscope (SEM) (TM3030, Hitachi, Tokyo, Japan).

#### Experimental Groups

The materials were divided into 3 experimental groups, as follows: **RMGIC**: resin-modified glass ionomer cement (Vitro Fil LC A3, DFL, Rio de Janeiro, Brazil); **NbG**: RMGIC + 10% experimental NbG (wt.%); and **45S5**: RMGIC + 10% 45S5 (wt.%).

RMGIC (Vitro Fil LC) is formed by a power portion (fluorine silicate, strontium, aluminum, calcium, charge, activators, and iron oxide) and liquid portion (2-hydroxyethyl methacrylate, an aqueous solution of polyacrylic and tartaric acids, benzoyl peroxide, and camphorquinone).

The former is hand-mixed with a dental spatula at a powder/liquid ratio of 1 spoon/2 drops. The powder is splinted in half, the first part of the powder is incorporated into the liquid and hand-mixed for 10 s, and then the remaining portion of powder is mixed for 10–15 s, following the manufacturer’s instructions. All preparation and measurements were carried out at room temperature.

For the manufacturing of the NbG and 45S5 groups, the bioactive materials were weighed using a precision balance before being incorporated in the RMGIC. Thereafter, 10% by weight of the RMGIC powder was removed from the flask, and the same proportion of the bioactive material (45S5 or NbG) was added to create the final experimental materials. The flasks containing the mixtures were shaken for 5 min using a vortex to standardize the material.

After incorporating the bioactive materials into the RMGIC, the manipulation of the materials followed the steps of the RMGIC group.

### 2.2. Physicochemical Evaluation

The alkalizing potential and fluoride release of the studied materials (RMGIC, NbG, and 45S5) were evaluated in an acidic medium (pH of 4, Merck S.A., São Paulo, Brazil). The acidic medium represents oral conditions that lead to tooth demineralization, contributing to the development of caries lesions.

Subsequently, the materials were applied to demineralized bovine dentin (São Luis, Brazil) to verify the material’s remineralizing potential, using a cross-sectional microhardness test.

#### Alkalizing Potential and Fluoride Release in an Acidic Medium (pH of 4)

To determine alkalizing potential and fluoride release, the materials were manipulated and inserted into a circular steel matrix (diameter: 10 mm, depth: 2 mm) to then be light-cured using a Bluephase N LED device (Ivoclar Vivadent, Schaan, Liechtenstein) at an intensity of 1200 mW/cm^2^ for 20 s, in accordance with the RMGIC Vitro Fil LC manufacturer’s specifications.

The prepared samples were initially used to verify the alkalizing potential (pH alteration). The medium (pH of 4.0) in which these samples were immersed was then evaluated for the release of fluoride.

For the alkalizing potential analysis, the samples were immersed in flasks containing solutions of 5 mL of deionized water with HCl, and the pH was adjusted to 4. The samples were stored in a bacteriological oven (Bunker Commercial Ltd.a, São Paulo, Brazil) at 37 ± 1 °C, and the pH of the solution was evaluated after 24 h as well as 7, 30, and 60 days.

The pH was measured three times at every evaluation time point by using a specific electrode (Quimis, Diadema, Brazil) that was calibrated and inserted into the flasks containing the solutions, and an average pH was calculated for each sample at each evaluation time point.

The fluoride release assessment was performed by using the solutions previously used for pH evaluation. The amount of fluoride released (ppm and mV) was recorded by using a fluoride-specific ion electrode (Quimis, Diadema, Brazil) coupled with a digital ion analyzer after 24 h as well as 7, 30, and 60 days.

The electrode was calibrated at each evaluation time point using standard solutions to form two analysis curves, each with 5 points. The reference points for the low-fluoride-concentration curves were 0.0625; 0.125; 0.25; 0.5; and 1 ppm, while those for the high-concentration curve were 6.25; 12.5; 25; 50; and 100 ppm.

The same volumes of the analyzed pH solutions were added to TISAB II (Thermo, Orion, São Paulo, Brazil) in the proportion of 0.5:0.5 mL (ratio of 1:1) [20] to adjust the pH of the solution, maintain its ionic strength, and allow the verification of the presence of fluorides.

### 2.3. Remineralization Potential

**Figure 1:**
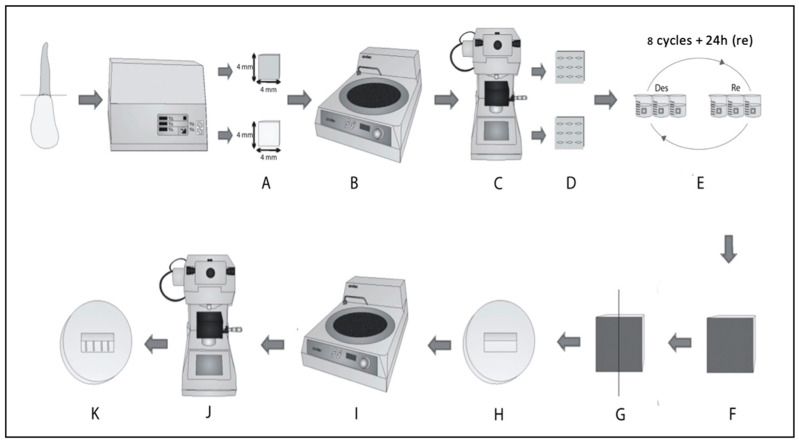
Methodology used. (**A**) Dental blocks; (**B**) buccal enamel removed using an Aropol-E polisher; (**C**) baseline microhardness of dentin was tested; (**D**) dentin blocks after indentations; (**E**) carious lesion induction; (**F**) view of the demineralized surface after pH cycles; (**G**) specimens sectioned in half (treatment X control); (**H**) dentin surface to be subjected to microhardness; (**I**) sample polishing for final microhardness measurement; (**J**) microhardness analysis after 24 h as well as 7 days, 30 days, and 60 days; and (**K**) final orientation of the indentations in the sample [23].

The dentin microhardness of thirty-six healthy bovine incisors (São Luis, Brazil) stored in distilled water was assessed to evaluate the remineralization potential of the experimental materials. The teeth were first embedded in PVC tubes with acrylic resin (Jet-clássico, São Paulo, Brazil) and sectioned using an IsoMet 1000 Precision Saw cutting machine (Buehler, Lake Bluff, NJ, USA) (Figure 1A) in the vestibulo-pulpal direction, so as to form enamel/dentin blocks (4 × 4 × 1 mm^3^) [21].

The dental blocks were then positioned on acrylic resin bases (Jet-clássico, São Paulo, Brazil) using sticky wax (NewWax, Rio de Janeiro, RJ, Brazil), and the buccal enamel was completely removed by using an Aropol-E polisher (Arotec, Cotia, Brazil). Dentin wear and regularization were carried out using #120, #600, and #1200 grit sandpaper (3M, Sumaré, Brazil), and the surfaces were polished using #2000 and #4000 grit sandpaper and felt disks with 2–4-micron polishing paste (Diamond Excel, FGM, Joinvile, Brazil) (Figure 1B).

#### 2.3.1. Surface Microhardness Analysis

The dentin blocks fixed on acrylic bases (Jet Acrylic Resin-Classic, Campo Limpo Paulista, Brazil) were positioned in the HMV-G20 Knoop microhardness tester (Shimadzu, Tokyo, Japan), and the baseline microhardness of dentin was tested using a 50 g load for 15 s (Figure 1C). Knoop microhardness is an ideal measurement for verifying the dentin hardness, relating this property to dental remineralizing aspects [22].

Three indentations were created on the surfaces of the samples in three rows at a spacing of 1000 μm from the center, and the mean value was calculated in order to obtain the final baseline microhardness (Figure 1D). Thereafter, the specimens were distributed into three experimental groups (n = 12) in a stratified manner according to the baseline microhardness values.

#### 2.3.2. Carious Lesion Induction

Buccal surface dentin was subjected to carious lesion induction cycles, while the other surfaces were isolated using an acid-fast varnish (Colorama Maybelline Ltda, São Paulo, Brazil). The artificial induction of dentinal caries was performed by using a previously validated protocol [22] consisting of exposing bovine dentin to an alternating pH cycle (Figure 1E).

The specimen was first immersed without agitation in a demineralizing solution composed of 2.2 mM CaCl_2_, 2.2 mM NaH_2_PO_4_, and 50 mM acetic acid (ISOFAR Ltd.a, Rio de Janeiro, Brazil) with the pH adjusted to 4.47 at 37 °C for 4 h on 8 consecutive days. Following this, it was immersed in a remineralizing solution composed of 1.5 mM CaCl_2_, 0.9 mM NaH_2_PO_4_, 130 mM KCl, and 20 mM buffer solution (ISOFAR Ltd.a, Rio de Janeiro, Brazil) with the pH adjusted to 7.0 at 37 °C for 20 h [23].

The pH cycle solutions were replaced every 4 days, and the specimens were washed in distilled water before each step to prevent contamination.

#### 2.3.3. Dentin Treatment

Following the induction of carious lesions, the specimens were randomly divided into three experimental groups (RMGIC; 45S5; and NbG) and then sectioned in half in the vestibulo-pulpal direction [21,22]. The buccal surface of one of the halves underwent treatment (Figure 1F–G) while the other half was stored in distilled water to serve as the control.

Each half of the dentine received treatment according to its group (RMGIC; 45S5; and NbG—the groups already mentioned above). In the case of this test, the materials were manipulated and then applied to the demineralized dentin surface.

To help the application of materials, a silicone matrix (Futura AD, Nova DFL, Rio de Janeiro, RJ, Brazil) was used to limit its quantity and standardize the thickness of the treatment. Light curing was carried out for 20 s and, following the handling and application of the materials, the demineralized dentin surfaces were stored in a bacteriological oven at 37 ± 1 °C.

After 24 h, the cross-sectional microhardness of the subsurface area of the treated dentin was measured. This area was first polished using sandpaper (#2000 and #4000) and felt disks with polishing paste (Diamond Excel, FGM, Joinville, Brazil; Figure 1H–I); the treated and untreated (control) groups then underwent microhardness analyses at distances of 10, 50, 100, and 200 µm from the external surface of the dentin. Readings were taken at 24 h as well as 7, 30, and 60 days after treatment (Figure 1J–K) [21].

### 2.4. Antimicrobial Activity

The antimicrobial activity of the bioactive materials was tested by using *S. mutans* UA159 biofilms (Campinas, São Paulo, Brazil) formed on the surface of the tested materials (n = 3). The samples were prepared by using a circular steel die (diameter: 6 mm, depth: 2 mm), and the materials were manipulated, inserted into the matrix, and light cured for 20 s.

All samples were sterilized using ethylene oxide (Merck, São Paulo, Brazil) before biofilm formation. Bacterial viability was quantified using colony-forming units (CFUs) and wet weight, while the acidogenicity of the biofilm was established via using the pH of the culture medium.

#### 2.4.1. Biofilm Growth

**Figure 2:**
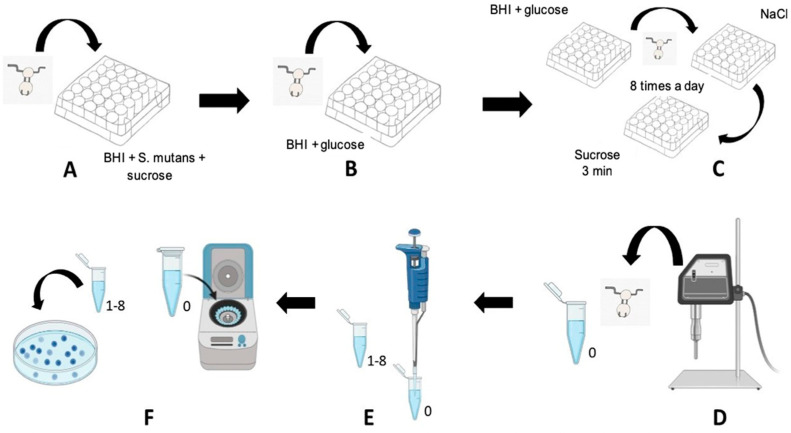
Methodology used. (**A**) Immersion of specimens in culture plates in a medium containing BHI + inoculum + 10% sucrose, for 8 h; (**B**) transfer of specimens to a new medium containing BHI + glucose every night; (**C**) sucrose baths: specimens washed with 0.01% NaCl and immersed in medium (BHI + 10% glucose) for 3 min, washed again in NaCl, and returned to the initial plate (8 times a day for 5 days); (**D**) sonication of specimens in an Eppendorf tube of 0.001% NaCl; (**E**) serial dilution of Eppendorf 0; and (**F**) centrifugation of Eppendorf 0, dilution of Eppendorf 0, and plating for CFU counting.

The formation of a salivary pellicle on the surfaces of the samples in the different experimental groups was carried out [24], following which the materials were placed in a sterile culture plate containing 2 mL of a medium composed of 15 μL of the inoculum (made with 15 μL of *S. mutans* UA159 in 10 mL of Brain Heart Infusion (BHI, KASVI, São José do Pinhais, Brazil) medium), 90% BHI medium (KASVI, São José do Pinhais, PR, Brazil), and 10% sucrose (St. Louis, MO, USA) for 8 h at 37 °C and 10% CO_2_ (Figure 2A).

After incubation, the blocks were transferred to new wells containing 2 mL of a new medium composed of 9 mL of BHI and 1 mL of glucose (St. Louis, MO, USA), where they remained overnight (Figure 2B). The following day, the samples were first washed three times in a 0.01% NaCl solution, exposed to sucrose baths for 3 min, and then immersed in a new culture medium containing 2 mL of BHI and glucose (Figure 2C). The samples were exposed to a 10% sucrose solution 8 times/day (8:00, 9:30, 11:00, 12:00, 13:30, 15:00, 16:00, and 17:30 h) for 3 min at a time. The same sequence was followed throughout, and the biofilms were washed 3 times in a 0.01% NaCl solution after each sucrose exposure. The medium was changed early in the morning and after the last exposure for the day, and the pH of each medium was measured to verify the acidogenicity of the biofilm [24].

#### 2.4.2. Biofilm Collection

After 120 h, the biofilms formed were collected separately by first washing the samples in 0.9% NaCl three times to remove loosely adherent material. Thereafter, they were immersed in Eppendorf tubes containing 1 mL of sterile saline solution (KASVI, São José do Pinhais, Brazil) and sonified at a power of 7 W for 30 s (Branson, Sonifier 50, Danbury, CT, USA) to remove the biofilms formed (Figure 2D) [24]. The samples were then removed from the Eppendorf tubes for the serial dilution of the suspension aliquots (Figure 2E), which were used to determine the colony-forming units (CFUs) and wet weight.

#### 2.4.3. Bacterial Viability Assessed Using Colony-Forming Units (CFUs) and Wet Weight

A 100 µL aliquot of the suspension was diluted in 1 mL of a sterile 0.9% NaCl solution serially to a concentration 10^−8^. Thereafter, two 20 µL drops of each serial dilution were inoculated onto BHI agar (BD, Sparks, MD, USA), and the plates were incubated for 48 h at 37 °C and 10% CO_2_. The colony-forming units (CFUs) were counted and expressed as CFU/mg of the wet weight of the biofilm [25].

After the serial dilution, the Eppendorf tubes containing the aliquots were centrifuged for 10 min to allow for the sedimentation of the inoculum, and the saline solution contained in the aliquot was aspirated. Centrifugation was repeated until all of the saline solution had been aspirated, and the Eppendorf tubes were then weighed using a high-precision balance. The values were recorded to determine the wet weight of each group.

### 2.5. Release of Calcium Ions

The material’s ability to release calcium ions in the *S. mutans* UA 159 biofilms was quantified after 24 and 120 h.

A measurement curve with 5 points (0.030; 0.050; 0.100; 0.150; and 0.200) was established to evaluate calcium ion release, and the culture medium of each group was analyzed by mixing it with Arsenazo III (Kovalent Ltd.a, Rio de Janeiro, Brazil) and subjecting it to an ELISA test using an Elx800 spectrophotometer (BioTek Instruments, Winooski, VT, USA). The analysis was repeated three times [26].

### 2.6. Statistical Analysis

The normality of alkalizing potential, fluoride as well as calcium ion release, and antimicrobial activity data was assessed by using the Levene test (*p* < 0.05). Scheffe’s two-way and post hoc ANOVA tests (*p* < 0.05) were used to evaluate the influence of time on changes in pH values, fluoride as well as calcium ion release, and biofilm acidogenicity. CFUs were evaluated by using an ANOVA with a post hoc Tukey test (*p* < 0.05).

The normality of data collected from the microhardness test was verified (Shapiro–Wilk, *p* > 0.05) and analyzed by using a mixed-design three-way ANOVA test (an independent test for the variables treatment and depth, and a repeated measure test for the variable time). A Student’s *t*-test for independent samples was used to compare the mean microhardness values of the experimental groups with their respective control groups at the same evaluation time and depth.

All statistical analyses were performed using IBM SPSS Statistic Base v.22.0 (Chicago, IL, USA), and the level of significance was set at *p* = 0.05.

## 3. Results

### 3.1. Distribution of Particle Size of the Resin-Modified Glass Ionomers

**Figure 3:**
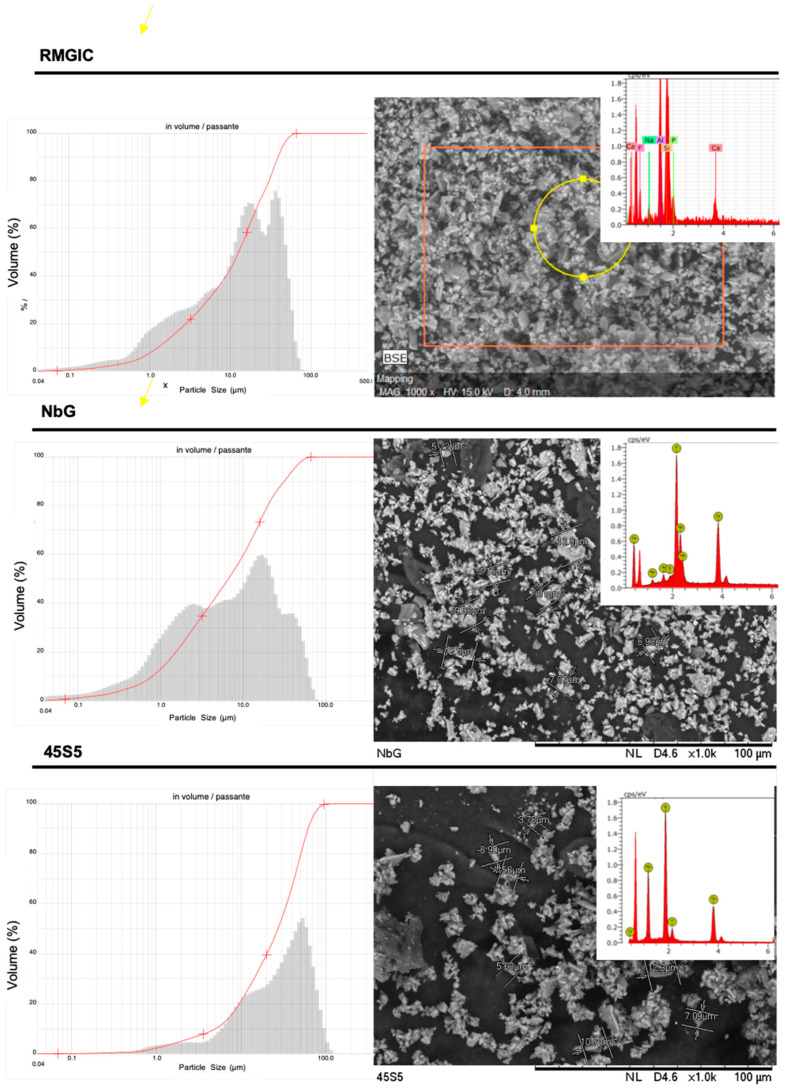
Particle size distribution of RMGIG (Vitro Fil), NbG, and 45S5, using a Malvern Zetasizer as well as SEM images.

The particle size and representative images of the materials studied, visualized by a scanning electron microscope (SEM), are shown in Figure 3. The presence of calcium particles in RMGIC was confirmed using EDS (Energy Dispersive X-Ray Spectroscopy). This material was enriched with NbG and 45S5 particles.

### 3.2. Physicochemical Evaluation: Variations in Alkalizing Potential and Fluoride Release

**Figure 4:**
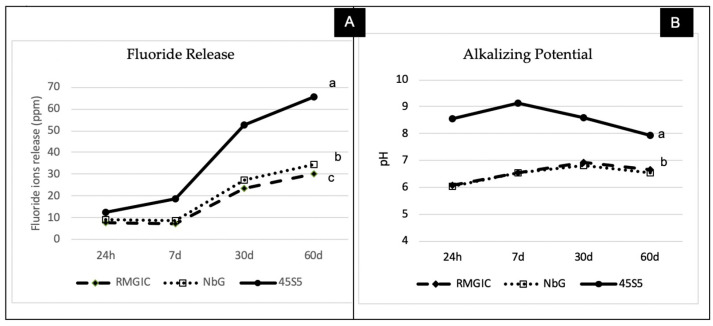
Mean values of the groups over time (24 h as well as 7, 30, and 60 days). (**A**) Release of accumulated fluoride ions. (**B**) pH change. Different letters represent significant statistical difference between materials (*p* < 0.05).

Due to the occurrence of demineralization in an acidic medium, the current study assessed changes in pH and the accumulated fluoride released by the experimental materials over a period of 60 days (Figure 4). The findings showed that the material versus time interaction was significant for alkalizing potential and fluoride ion release (*p* < 0.001), with the latter exhibiting a significant increase over the 60 days (*p* < 0.001; Figure 4A). In an acidic medium, the 45S5 group exhibited greater alkalizing potential and ability to release fluoride ions compared to the NbG and RMGIC groups (*p* < 0.001) (Figure 4B). The highest pH values were recorded after 7 and 30 days (*p* < 0.001) in all groups at all evaluation time points.

### 3.3. Cross-Sectional Microhardness (KHN)

**Figure 5:**
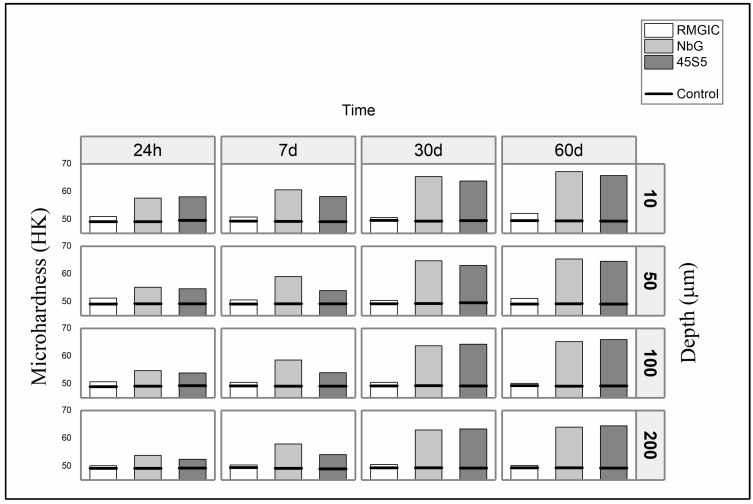
Mean microhardness values for the experimental groups (columns) and their respective controls (rows), evaluated according to the evaluation time and preparation depth.

The treatment method (*p* < 0.001), time point (*p* < 0.001), and depth (*p* < 0.001) were seen to influence the hardness of demineralized dentin (Figure 5). The association between the material and time variables was significantly different (*p* < 0.001), as was the interaction between time and depth (*p* < 0.001).

The microhardness was significantly higher at a depth of 10 µm (*p* < 0.001), and the values were seen to vary with depth over time (*p* < 0.001). The mean microhardness values were influenced by the type of material used at the time of evaluation (*p* < 0.001).

Untreated demineralized dentin (control groups) exhibited stable mean microhardness values over the 60 days, and the *t*-test for independent samples showed that the treatment material (RMGIC, 45S5, and NbG) differed significantly from the control group when compared at the same evaluation time point and depth (*p* < 0.05). The type of material used had a significant effect on the mean microhardness value (*p* < 0.001). It is also worth mentioning that the *p*-value of the difference between RMGIC and the other groups was <0.001, while that between the 45S5 and NbG groups was 0.040.

### 3.4. Biofilm Acidogenicity and Bacterial Viability

**Figure 6:**
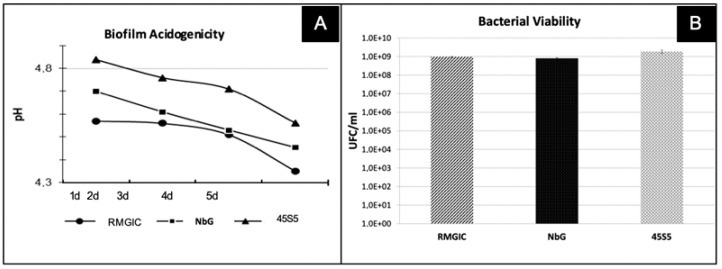
Antimicrobial activity. (**A**) Mean values of biofilm acidogenicity. (**B**) Mean values of wet weight (CFU/mL) and amount of soluble proteins, according to the treatment for the experimental groups.

A significant difference in pH change was observed between the various groups and at the different time points (2, 3, 4, and 5 days; *p* < 0.001), with the highest pH values being observed in the 45S5 group (*p* < 0.001) (Figure 6A).

Bacterial viability in the biofilm, normalized by wet weight (Figure 6B), did not differ between the groups (*p* > 0.05).

### 3.5. Calcium Ion Release

**Figure 7:**
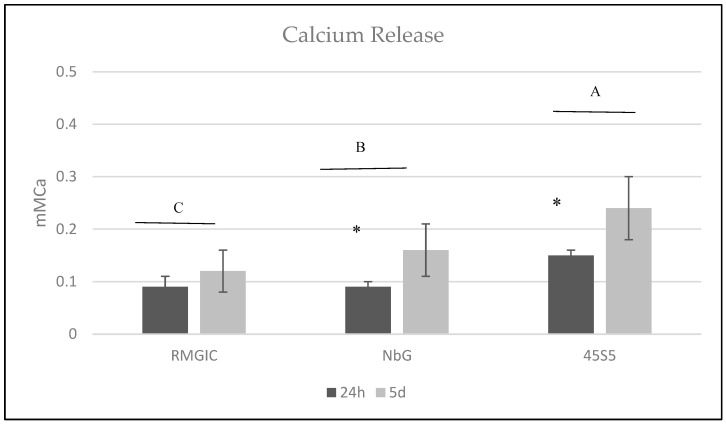
Mean values of accumulated calcium ion release for different materials. Different capital letters (A, B and C) represent the statistical difference between materials (*p* < 0.05). The presence of symbols (*) represents the statistical difference between times (24 and 120 h) (*p* < 0.05).

In the *S. mutans* UA159 biofilms, a statistically significant difference in the release of calcium ions was observed between the different groups and evaluation time points (*p* < 0.001). The highest calcium release was observed after 5 days (Figure 7), and the 45S5 exhibited greater calcium release under these conditions when compared to the other groups (*p* < 0.001) (Figure 7).

## 4. Discussion

Clinical studies have shown that the maintenance of affected dentin can promote the greater conservation of tooth structure and increase clinical [27] as well as radiographic success rates over time [2]. Within this context, the present study evaluated the influence of the incorporation of bioactive glasses (45S5 and NbG) in resin-modified glass ionomer cement at a concentration of 10% on dentin remineralization. The results showed that 45S5, a bioactive material, exhibited the greatest potential for alkalizing acidic as well as microbial culture media and also released the greatest amount of fluoride and calcium ions. The cross-sectional microhardness test showed the highest values for 45S5 and NbG. Therefore, all of the hypotheses being tested in this study were rejected.

The development of new bioactive materials that promote dental structure remineralization is necessary as the presence of an acidic environment supports the development of caries [28]. Satisfactory results with regard to the remineralization of demineralized dentin can be observed after 30 to 60 days when significant pulp repair and formation of reparative dentin [29] have occurred, thus reducing the recurrence of carious lesions [30].

The evaluation of the alkalizing potential of 45S5 and NbG, as well as their ability to release fluoride ions, was carried out, as these outcomes play an important role in dentin remineralization. The presence of fluoride ions during the remineralization process is important as it limits caries depth and progression [31] by inhibiting the demineralization of the dental structure. The incorporation of 45S5 at a concentration of 10% (by weight) in the resinous material was examined, as it has been reported that the chemical and mechanical properties of the material are not affected at this concentration [32].

The results of this study showed that the bioactive materials studied (45S5 and NbG) exhibited greater alkalinization of the medium and fluoride release (Figure 4), corroborating previous findings from the literature [23,33]. The pH analysis showed that the 45S5 group exhibited greater alkalizing potential in an acidic medium, and this can contribute to remineralization [14] as well as the formation of precipitates in dentin [34]. This finding can be attributed to the rapid release of ions of a basic nature (such as sodium) and the capture of hydrogen protons, which are incorporated into the surface of the particles [35]. This behavior can decrease the maintenance of the “critical pH”, thus limiting the demineralization of the tooth structure and the risk of caries progression [28].

It has been suggested that the incorporation of HEMA into GIC is capable of decreasing fluoride ion release as it alters the acid–base reaction and leads to the formation of a weaker gel network [20]; however, no consensus has been reached about this in the literature, with some studies demonstrating greater fluoride ion release in RMGIC [8]. The findings of the current study showed that the incorporation of 45S5 into RMGIC potentiated the release of fluoride ions from this material.

In the context of minimal intervention treatment measures, dentin remineralization following selective caries removal can promote the greater longevity of the dentin–resin interface [36]. In this study, the remineralizing potential of the materials was evaluated by using the microhardness test, as reports that Ca:P rates can be correlated with microhardness values are accepted for caries lesions [3]. Microhardness values can predict mineral content. Laboratory studies have used microhardness data in the Featherstone et al. 1983 equation to calculate percentage mineral volume [37], as a correlation has been found between microhardness profiles and mineral content in human enamel and dentin (r = 0.92). The RMGIC group showed no changes in dentin microhardness, while higher values were observed in the NbG and 45S5 groups (Figure 5). This increase can be attributed to the fact that bioactive materials such as 45S5 can help restore the mineralization pattern and stimulate hydroxyapatite deposition, generating a stable bond with dentin [38]. A previous study [17] reported that this RMGIC/45S5 association showed a peak at 670 cm^−1^ (Si–OH), associated with NbG bioactive glass, and 570 cm^−1^ (phosphate: PO_4_^−3^) in an FTIR/ATR analysis. This can, in turn, affect the mechanical characteristics of the dental structure by increasing dentin microhardness [17]. Furthermore, bioactive materials such as 45S5 and NbG have been shown to exhibit greater interaction with dentin in the first 24 h and maintain this increased microhardness over time [39], and this was in accordance with the findings of the current study.

As increased pH [40] and the release of ions from bioactive materials can inhibit bacterial growth [41], the present study also evaluated the antimicrobial activity of the study materials, as it can contribute to the limitation of carious lesion development. Thus, biofilms formed by *Streptococcus mutans* UA159, the main caries-related species that represents the dominant *Streptococcus genus* in dental plaque biofilms, were analyzed [42].

However, the findings showed that the wet weights of the biofilms formed on the bioactive materials (45S5 and NbG) were similar to those seen in the RMGIC control group (Figure 6). Yoshihara et al., 2017 [41], also found that, despite the release of ions from bioactive materials, bacterial growth was not inhibited and the increase in surface roughness promoted biofilm formation [41], and this corroborated the findings of this study. Although 45S5 and NbG exhibited satisfactory alkalizing potential in an acidic medium (pH of 4.0) in the presence of acidogenic and aciduric bacteria (*S. mutans*), they did not present superior results concerning bacterial growth when compared to the control group (RMGIC).

Previous studies report satisfactory antibacterial activity by 45S5 and NbG bio-glasses [11,16]; however, the methodologies applied in these studies differ from the current one, where a sample of RMGIC incorporated with 10% of 45S5 was analyzed. In contrast, Bauer et al. (2019) [11] used a suspension containing 20% of 45S5, suggesting that a solid sample may have a lower potential for antimicrobial activity compared to materials presented in powder or suspension form [11].

The ability to release calcium ions in a microbiological medium was higher in the 45S5 group, although all groups exhibited significant release over time. Previous literature demonstrates that nanoparticulate 45S5 bioglass is a source of calcium and silica ions [34] that induces dentin remineralization after 30 days of treatment [34], and this corroborated the findings of this study. The NbG group exhibited calcium release similar to the RMGIC group, and these results may reflect the delayed ionic release capacity of NbG [26]. Carneiro et al. (2018) [43] showed that the peak of calcium release by NbG was reached after 90 days of evaluation [43]. Thus, in a short-period evaluation, 45S5 presents better performance in regard to physicochemical properties than NbG; however, the stability of NbG can potentially guarantee the beneficial effect of the material for a longer period of time, according to the evaluation values of the cross-sectional microhardness of this study. The present study restricted the calcium release assessment period to 5 days to correspond with the microbiological test protocol. Perhaps a longer period was necessary in order to be able to elucidate the total release of calcium ions from NbG into the medium. The maintenance of pH over time can be attributed to the chemical stability of NbG [43].

In this way, it is possible to verify that the incorporation of bioactive agents (45S5 and NbG) into RMGIC can assist in the remineralization of demineralized dentin through the release of fluoride ions and the alkalization of the medium [28]; however, further research is necessary to complement the findings of this study.

## 5. Conclusions

The improvement of the physicochemical properties of resin dental materials, such as RMGIC, could be observed with the addition of bioactive particles such as NbG and 45S5. Under the influence of the incorporation of these bioactive particles, the release of fluorine was increased. In a microbiological challenge condition there was a greater tendency to decrease the acidogenicity of the biofilm and increase the release of calcium, these values being more expressive for 45S5. Within the limitations of this in vitro study, it was also observed that the incorporation of NbG and 45S5 increased the cross-sectional microhardness of dentin, which represents the potential of these materials to help the remineralization of demineralized dental structures. These aspects indicate that the enrichment of resinous materials with bioactive materials can contribute to greater success in paralyzing caries lesions.

## Data Availability

Not applicable.
